# Explainable machine learning for identifying high-risk Alzheimer's disease and key health characteristics among community-dwelling adults

**DOI:** 10.3389/fpubh.2026.1943300

**Published:** 2026-10-12

**Authors:** Huanhuan Zhao, Bai Qin, Zengli Zou, Pingjing Wen

**Affiliations:** 1 School of Public Health and Management, Guangxi University of Chinese Medicine, Nanning, China; 2 Public Health Division, The Third People's Hospital of Nanning, Nanning, China

**Keywords:** Alzheimer's disease, community-dwelling adults, machine learning, risk assessment, SHAP

## Abstract

**Objective:**

To develop an interpretable machine learning model to identify individuals with AD8-defined high-risk status for Alzheimer's disease among community-dwelling adults, identify key health characteristics and their association patterns with high-risk status, and to inform community-based cognitive health promotion.

**Methods:**

Based on health survey data from 6,267 community-dwelling adults, AD8-defined high-risk status for Alzheimer's disease was assessed using the Alzheimer's Disease Screening Scale (AD8). LASSO logistic regression, random forest, and decision tree models were constructed, and SHapley Additive exPlanations (SHAP) was used to analyze the importance of key health characteristics and their association patterns.

**Results:**

A total of 6,267 community-dwelling adults were included, of whom 1,454 (23.2%) were classified into the AD8-defined high-risk group. The random forest model showed the highest discriminative performance among the three models. SHAP analysis indicated that fruit intake, age, snoring, mean grip strength, and sleep duration were important health characteristics associated with AD8-defined high-risk status for Alzheimer's disease. SHAP dependence plots further revealed a graded negative association between fruit intake and high-risk status, a U-shaped association for sleep duration, a U-shaped association for mean grip strength, and a positive graded association between snoring frequency and high-risk status.

**Conclusions:**

Random forest combined with SHAP identified key health characteristics associated with AD8-defined high-risk status among community-dwelling adults and revealed non-linear association patterns involving health behaviors and physical function. These findings may provide useful information for community-based risk stratification and targeted cognitive health promotion.

## Introduction

Alzheimer's disease (AD) is the most common neurodegenerative disease and the leading cause of dementia (1). With the ongoing intensification of global population aging, the number of AD patients continues to rise. This trend has made AD a major public health issue that threatens the health and quality of life of older adults. The World Health Organization projects that the number of dementia patients worldwide will continue to increase rapidly in the coming decades, further aggravating the disease burden and imposing tremendous pressure on patients' families, healthcare systems, and socioeconomic development (2). Given the absence of curative treatments, disease prevention and control strategies have gradually shifted toward early intervention. Early identification of high-risk populations and implementation of health interventions are considered important approaches to delaying cognitive decline and reducing the disease burden (3). Therefore, exploring the influencing factors of high-risk status for Alzheimer's disease in community populations and establishing a scientific and convenient early identification strategy are of great significance for advancing community-based cognitive health management.

The occurrence and progression of Alzheimer's disease are influenced by multiple factors and are characterized by a distinctly multifactorial, multilevel, and complex nature. Existing research indicates that demographic factors such as age and sex are important background factors for disease onset, with age currently recognized as the most established non-modifiable risk factor (1); cardiovascular and metabolic abnormalities such as hypertension and obesity can promote neurodegenerative changes through mechanisms including cerebrovascular injury, chronic inflammation, and metabolic dysregulation (4); lifestyle factors such as sleep abnormalities and unhealthy diet are closely associated with cognitive decline (5); and physical function indicators such as grip strength can reflect the degree of aging of the body, with declines in these indicators also considered to be closely related to cognitive decline and the risk of dementia (6). These risk factors do not operate in isolation; rather, they jointly influence cognitive function through complex interactions, which may involve non-linear relationships and synergistic effects. Therefore, comprehensively identifying high-risk populations in the community based on multidimensional health characteristics aligns better with the characteristics of Alzheimer's disease onset than single risk factor analysis, and also better meets the needs of precision intervention in public health.

Traditional epidemiological studies have primarily used classical statistical methods to analyze risk factors for Alzheimer's disease, but they have certain limitations in handling multidimensional variables, complex non-linear relationships, and variable interactions. In recent years, machine learning algorithms such as random forest, support vector machine, and gradient boosting tree have shown certain advantages in dealing with complex variable relationships (7). However, existing studies mostly focus on model classification performance, and the interpretation of the model decision-making process and the mechanisms of key health characteristics remains insufficient. SHapley Additive exPlanations (SHAP), as a widely used Explainable Artificial Intelligence (XAI) method, can interpret machine learning models at both the global and local levels. It can not only quantify the contribution of each feature to the model prediction results but also reveal potential non-linear relationships between features and prediction results, thereby improving the transparency and interpretability of the model (8) and providing more transparent and credible explanations for machine learning models.

Currently, the early identification of high-risk status for Alzheimer's disease among community-dwelling adults still faces the challenge of simultaneously balancing classification performance and model interpretability. Meanwhile, studies on identifying key modifiable health features and their association patterns based on routine community health indicators remain relatively limited. Therefore, this study used health survey data from community-dwelling adults to construct LASSO logistic regression, random forest, and decision tree models, select the model with the best overall performance, and integrate the SHapley Additive exPlanations (SHAP) method to identify key health features associated with high-risk status for Alzheimer's disease and further reveal their association patterns with the high-risk status, so as to provide a scientific basis for community-based cognitive health screening, health promotion, and precise intervention.

## Methods

### Participants

This cross-sectional study was conducted in Nanning City, Guangxi Zhuang Autonomous Region, China, between October 2025 and February 2026. A cluster sampling strategy was used to recruit permanent residents aged 18 years and older from six primary healthcare institutions in Xixiangtang District, Nanning: Shangyao Community Health Service Center, Xinyang Zhongxing Community Health Service Center, Xixiangtang Township Health Center, Shibu Township Health Center, Jinling Central Township Health Center, and Tanluo Central Township Health Center, as part of a joint chronic disease survey. A total of 6,856 individuals participated in the initial survey. After excluding 117 participants with missing AD8 or key health characteristic data and 472 participants with abnormal data, 6,267 community-dwelling adults were included in the final analysis. Abnormal data were defined as logically impossible age–education combinations, physiologically implausible physical examination values, or internally inconsistent self-reported health information.

Inclusion criteria included: (1) age ≥ 18 years; (2) not diagnosed with Alzheimer's disease at the time of the survey; and (3) complete information on Alzheimer's disease risk assessment and major health characteristic variables. This study was approved by the ethics committee, and written informed consent was obtained from all study participants.

### Variables

#### Outcome variable

The outcome variable was AD8-defined high-risk status for Alzheimer's disease. In this study, the Alzheimer's disease screening scale AD8 (Ascertain Dementia 8) (9) was used to perform cognitive function screening on the study participants. The AD8 is a simple, reliable cognitive function screening tool primarily used to identify individuals at risk of cognitive decline and has been widely applied in early screening for cognitive impairment in community populations and primary care settings. Because Alzheimer's disease is the most common cause of dementia (10), the AD8 is also frequently used for the initial identification of high-risk populations for Alzheimer's disease in the community.

Based on the AD8 scores, the study participants were divided into a low-risk group (AD8 < 2 points, assigned a value of 0) and an AD8-defined high-risk group (AD8 ≥ 2 points, assigned a value of 1), which served as the categorical outcome variable for the machine learning models.

#### Predictor variables

Based on previous epidemiological studies, community health survey content, and data completeness, this study selected health characteristics related to cognitive function as candidate predictor variables, and a total of 15 indicators were included for machine learning model construction. To fully leverage machine learning's capacity for handling complex non-linear relationships and variable interactions, we did not pre-screen variables using univariate analysis. Instead, all candidate variables were simultaneously incorporated into model training, allowing the model to automatically learn each variable's contribution to classifying high-risk AD status.

The predictor variables encompass four dimensions.

(1) Demographic characteristics: age (years) and sex (male = 1, female = 0).
(2) Cardiovascular and physical health indicators: systolic blood pressure (SBP, mmHg), diastolic blood pressure (DBP, mmHg), heart rate (beats/min), body mass index (BMI, kg/m^2^), and waist-hip ratio (WHR).
(3) Physical function indicator: mean grip strength (kg). Grip strength was measured using an electronic dynamometer, with two measurements taken for each hand. The mean of the four measurements was used as the final value.
(4) Lifestyle indicators: sleep duration (h), snoring, and intake frequency of fruits, nuts, milk, seafood, and soy products.

All the above predictor variables were obtained from community health questionnaires and physical examinations. They are characterized by easy access, low cost, and ease of roll-out, making them suitable for the practical application scenario of early screening for high risk of Alzheimer's disease in community populations.

### Statistical analysis

All statistical analyses were performed using R software (version 4.5.3). Continuous variables were expressed as mean ± standard deviation (x ± s), and comparisons between groups were conducted using the independent samples *t*-test. Categorical variables were expressed as frequencies and percentages, and comparisons between groups were conducted using the χ^2^ test. A two-tailed *P* < 0.05 was considered statistically significant.

All study subjects were randomly divided into a training set (70%) and a testing set (30%). Five-fold cross-validation was performed entirely within the training set for hyperparameter tuning and internal validation, and the testing set was held out and used only for the final evaluation of model performance. LASSO logistic regression, random forest (RF), and decision tree (DT) models were constructed. Given the imbalance between the high-risk and low-risk groups, class weights were applied to all three models during training to mitigate the impact of class imbalance. Class weights were calculated as the inverse of class frequencies in the training set and normalized to a mean of 1. Specifically, the mtry parameter was optimized for random forest, the penalty parameter (λ) for LASSO logistic regression was selected using the one-standard-error rule, and the complexity parameter (cp) was optimized for decision tree. The final hyperparameter settings and class weights are summarized in Supplementary Table S1. Subsequently, the final models were reconstructed based on the optimal parameters, and their classification performance was evaluated on the testing set. Classification thresholds were determined using the Youden index and applied consistently to calculate accuracy, sensitivity, specificity, F1 score, and the Matthews correlation coefficient (MCC). The classification performance of the models was assessed using the receiver operating characteristic (ROC) curve and the area under the curve (AUC), in conjunction with accuracy, sensitivity, specificity, F1 score, and MCC. Given the study objective of exploring potentially non-linear relationships and interactions among multidimensional health characteristics, the model with competitive discrimination and the capacity to capture non-linear patterns was selected for subsequent SHAP-based interpretation.

The SHapley Additive exPlanations (SHAP) method was adopted to interpret the optimal model, with variable importance assessed via mean absolute SHAP values. SHAP values were computed on a random subset of 500 participants from the training set. SHAP summary plots and SHAP dependence plots were generated to analyze the contributions of key health features to the high-risk state of Alzheimer's disease and their associated patterns. SHAP dependence curves for continuous variables were fitted using locally weighted scatterplot smoothing (LOESS) with a 95% confidence interval. For ordinal variables, boxplots were used to show the distribution of SHAP values across categories. Ordinal variables, including intake frequency of fruits, nuts, milk, seafood, and soy products, and snoring frequency, were coded as ordered numeric variables (1 = never, 2 = rarely, 3 = sometimes, 4 = often, 5 = daily).

## Results

### Baseline characteristics of the study population

A total of 6,267 community-dwelling adults were included in this study, consisting of 4,813 (76.8%) in the low-risk group for Alzheimer's disease and 1,454 (23.2%) in the high-risk group. Statistically significant differences were observed between the two groups in demographic characteristics, cardiovascular health indicators, physical function, and certain lifestyle factors (Table 1). Compared with the low-risk group, the high-risk group was older, had a higher proportion of females, and exhibited lower systolic blood pressure, diastolic blood pressure, average grip strength, and sleep duration (all *P* < 0.001).

**Table 1:** Baseline characteristics of the study participants.

Variables	Total (*N* = 6,267)	Low risk (*N* = 4,813)	High risk (*N* = 1,454)	*P*-value
Age (years)	51.41 (13.73)	50.81 (13.69)	53.38 (13.67)	< 0.001
Sex				< 0.001
Female	4,184 (66.8)	3,122 (64.9)	1,062 (73.0)	
Male	2,083 (33.2)	1,691 (35.1)	392 (27.0)	
SBP (mmHg)	126.75 (19.59)	127.14 (19.74)	125.44 (18.90)	0.003
DBP (mmHg)	80.22 (11.06)	80.55 (11.26)	79.11 (10.35)	< 0.001
BMI	23.64 (3.40)	23.66 (3.40)	23.58 (3.38)	0.415
WHR	0.86 (0.08)	0.86 (0.08)	0.86 (0.08)	0.485
Heart rate	76.99(11.41)	77.37(11.55)	75.75(10.84)	< 0.001
Sleep_hour (h)	6.92 (1.34)	6.96 (1.32)	6.77 (1.39)	< 0.001
Grip (kg)	18.39 (7.28)	18.72 (7.37)	17.31 (6.84)	< 0.001
Fruit				< 0.001
Never/almost never	284 (4.5)	194 (4.0)	90 (6.2)	
1–3 times/month	451 (7.2)	330 (6.9)	121 (8.3)	
1–3 times/week	1,590 (25.4)	1,180 (24.5)	410 (28.2)	
4–6 times/week	1,178 (18.8)	885 (18.4)	293 (20.2)	
Daily	2,764 (44.1)	2,224 (46.2)	540 (37.1)	
Nut				< 0.001
Never/almost never	2,492 (39.8)	1,822 (37.9)	670 (46.1)	
1–3 times/month	1,736 (27.7)	1,374 (28.5)	362 (24.9)	
1–3 times/week	1,203 (19.2)	979 (20.3)	224 (15.4)	
4–6 times/week	540 (8.6)	423 (8.8)	117 (8.0)	
Daily	296 (4.7)	215 (4.5)	81 (5.6)	
Milk				< 0.001
Never/almost never	2,325 (37.1)	1,709 (35.5)	616 (42.4)	
1–3 times/month	1,294 (20.6)	1,017 (21.1)	277 (19.1)	
1–3 times/week	1,359 (21.7)	1,080 (22.4)	279 (19.2)	
4–6 times/week	625 (10.0)	492 (10.2)	133 (9.1)	
Daily	664 (10.6)	515 (10.7)	149 (10.2)	
Seafood				0.013
Never/almost never	1,840 (29.4)	1,365 (28.4)	475 (32.7)	
1–3 times/month	1,951 (31.1)	1,503 (31.2)	448 (30.8)	
1–3 times/week	1,628 (26.0)	1,290 (26.8)	338 (23.2)	
4–6 times/week	675 (10.8)	521 (10.8)	154 (10.6)	
Daily	173 (2.8)	134 (2.8)	39 (2.7)	
Soybean products intake				< 0.001
Never/almost never	1,720 (27.4)	1,235 (25.7)	485 (33.4)	
1–3 times/month	1,425 (22.7)	1,096 (22.8)	329 (22.6)	
1–3 times/week	1,991 (31.8)	1,571 (32.6)	420 (28.9)	
4–6 times/week	723 (11.5)	572 (11.9)	151 (10.4)	
Daily	408 (6.5)	339 (7.0)	69 (4.7)	
Snore				< 0.001
Never/almost never	2,530 (40.4)	1,986 (41.3)	544 (37.4)	
1–3 times/month	1,608 (25.7)	1,254 (26.1)	354 (24.3)	
1–3 times/week	732 (11.7)	554 (11.5)	178 (12.2)	
4–6 times/week	392 (6.3)	301 (6.3)	91 (6.3)	
Daily	1,005 (16.0)	718 (14.9)	287 (19.7)	

Continuous variables are expressed as mean ± standard deviation; categorical variables are presented as *n* (%).

### Performance comparison of machine learning models

Three machine learning models were constructed: LASSO logistic regression, random forest (RF), and decision tree (DT). The classification performance of each model is shown in Table 2, and the ROC curves are presented in Figure 1. RF achieved the highest AUC (0.647), followed by LASSO (0.606) and DT (0.560). At the Youden-optimal cutoff, RF also showed the highest MCC (0.183), while the other classification metrics varied across the three models. The difference in AUC between LASSO and RF was statistically significant according to the DeLong test (LASSO – RF: difference = −0.0408, 95% CI: −0.0698 to −0.0118, *P* = 0.006). Given its comparatively better discriminatory performance, RF was selected for subsequent SHAP analysis.

**Table 2:** Performance comparison of three machine learning models.

Model	AUC	Cutoff	Accuracy	Sensitivity	Specificity	F1	MCC
Lasso	0.606	0.236	0.614	0.541	0.636	0.394	0.152
Random forest	0.647	0.239	0.625	0.571	0.642	0.414	0.183
Decision tree	0.560	0.234	0.566	0.530	0.577	0.362	0.090

**Figure 1:**
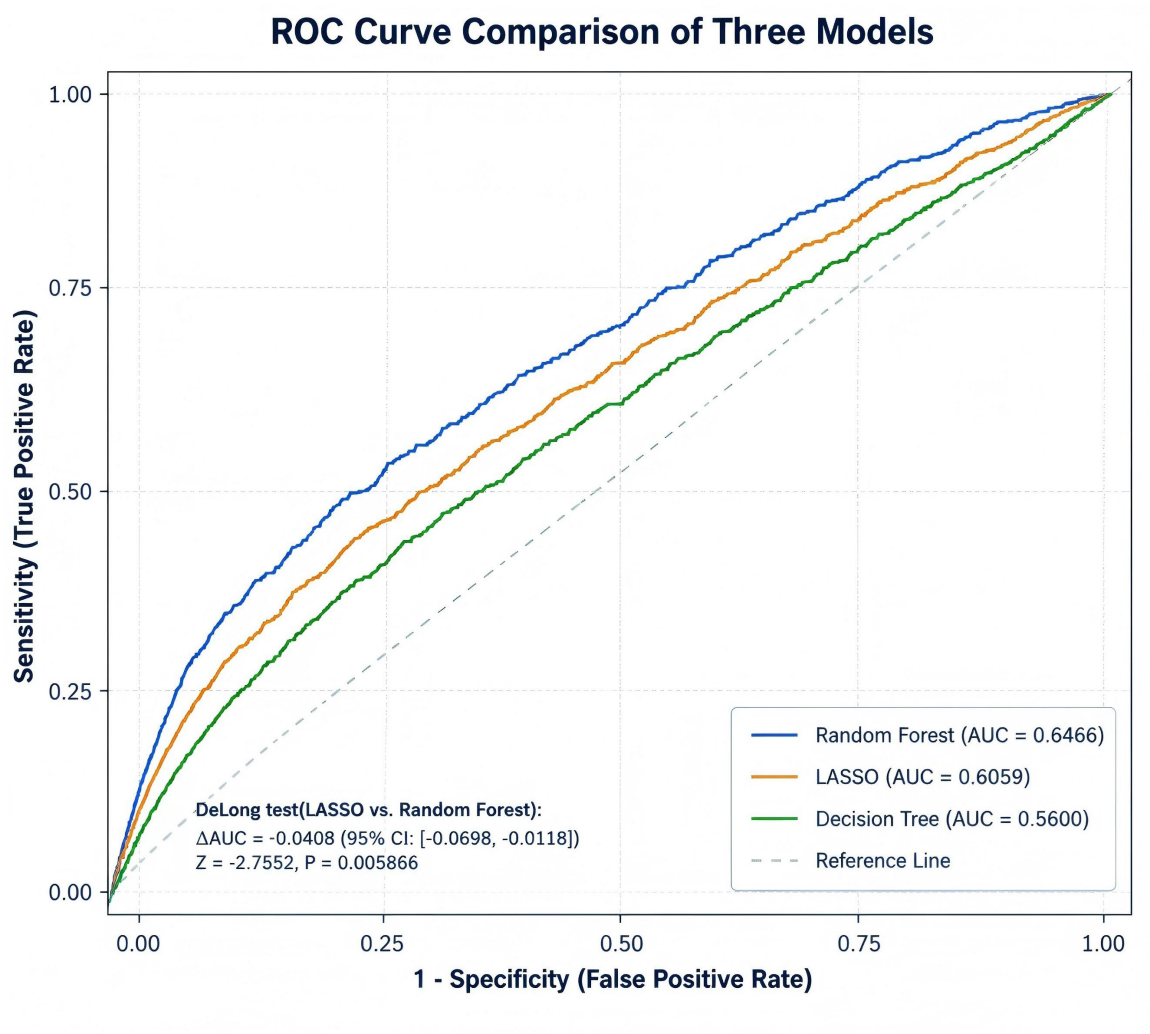
ROC curve.

### SHAP-based feature importance analysis

The SHapley Additive exPlanations (SHAP) method was used to perform interpretability analysis on the random forest model, and a SHAP feature importance bee swarm plot (Figure 2) was generated. The contribution of each variable to the model classification results was assessed based on the mean absolute SHAP value. The 15 predictor variables were ordered from high to low according to their mean absolute SHAP values (Table 3).

**Figure 2:**
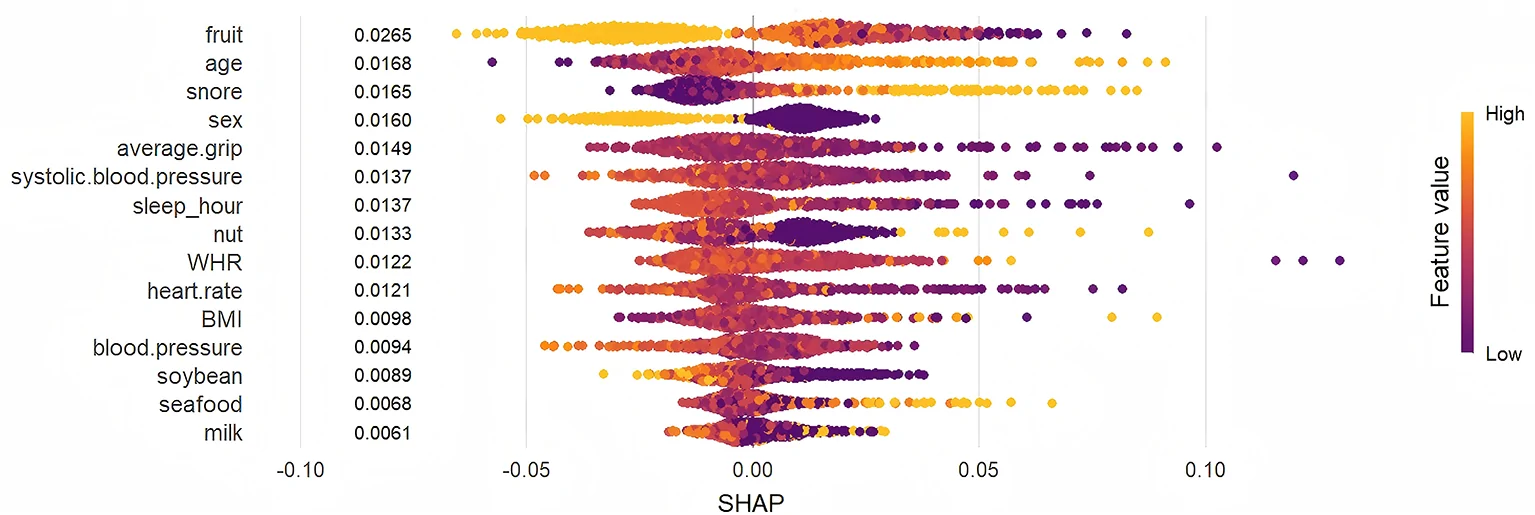
SHAP summary plot.

**Table 3:** Ranking of feature importance based on SHAP values.

Rank	Variable	Average SHAP value	Importance
1	Fruit	0.0265	High
2	Age	0.0168	High
3	Snore	0.0165	High
4	Sex	0.0160	High
5	Grip	0.0149	High
6	SBP	0.0137	Medium
7	Sleep_hour	0.0137	Medium
8	Nut	0.0133	Medium
9	WHR	0.0122	Medium
10	Heart rate	0.0121	Medium
11	BMI	0.0098	Low
12	DBP	0.0094	Low
13	Soybean	0.0089	Low
14	Seafood	0.0068	Low
15	Milk	0.0061	Low

Based on the quantiles of the average SHAP values, variable importance is classified into three levels: high, medium, and low.

The SHAP analysis results showed that fruit intake (0.0265) contributed the most to model classification, followed by age (0.0168), snoring (0.0165), sex (0.0160), average grip strength (0.0149), systolic blood pressure (0.0137), sleep duration (0.0137), nut intake (0.0133), waist-to-hip ratio (0.0122), and heart rate (0.0121). The contributions of BMI, diastolic blood pressure, and the intake of soy products, seafood, and dairy were relatively low. The bee swarm plot revealed that older age, female sex, frequent snoring, and lower intakes of fruit and nuts, as well as lower grip strength, were associated with higher SHAP values, whereas increased fruit and nut intake, longer sleep duration, and higher grip strength corresponded to lower SHAP values, suggesting that these health characteristics are closely associated with the high-risk state for Alzheimer's disease identified by the model. The bee swarm plot also demonstrated the direction of influence of different variable values on model classification results and the heterogeneity among individuals, providing a basis for further analysis of the association patterns between key health characteristics and the high-risk state for Alzheimer's disease.

### SHAP dependence analysis of key health characteristics

To further analyze the association patterns between key health characteristics and a high-risk state of Alzheimer's disease, SHAP dependence plots for age, fruit intake, sleep duration, snoring, and average grip strength were further generated (Figure 3).

**Figure 3:**
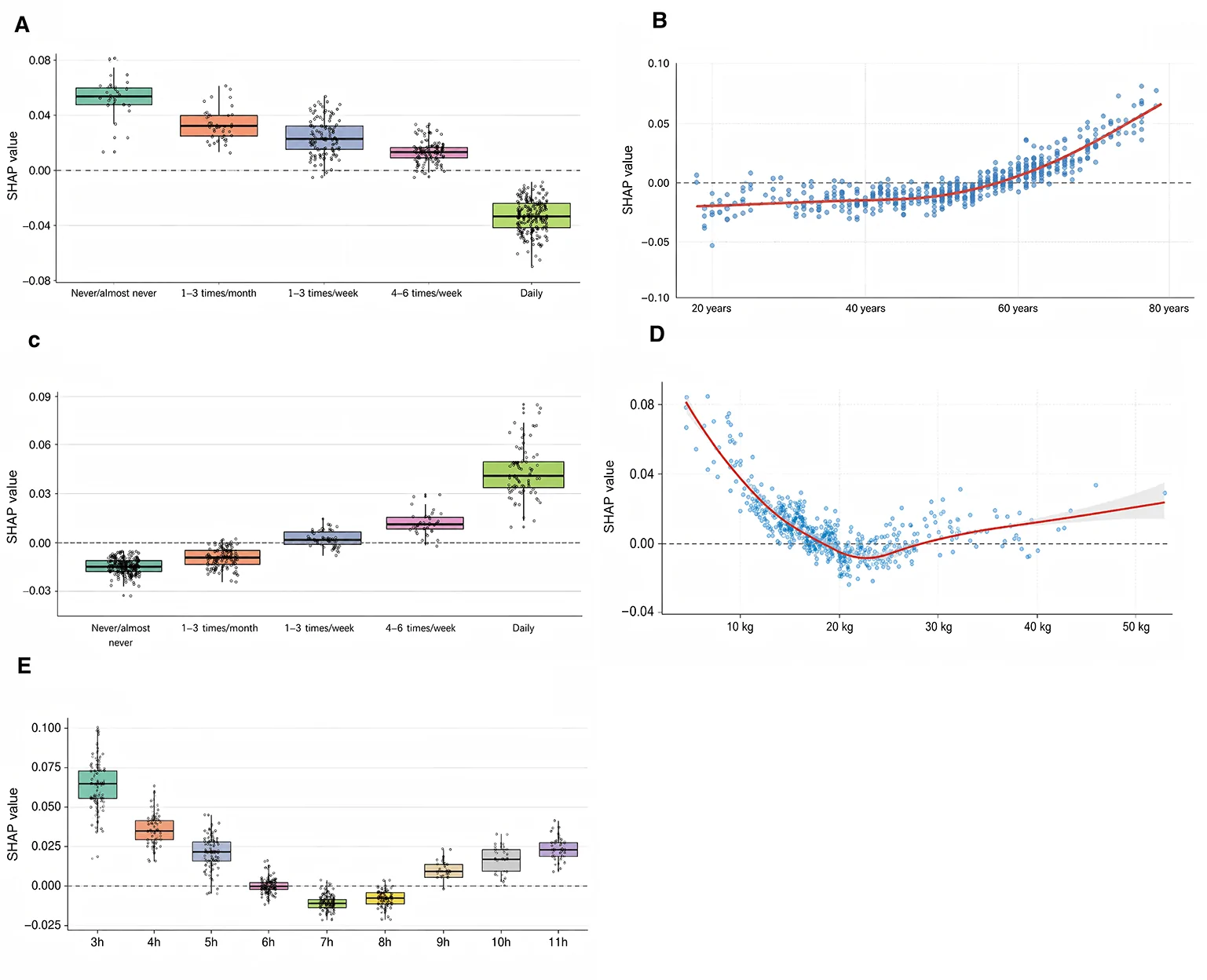
SHAP dependence plots. **(A)** SHAP value distribution of fruit intake frequency on AD risk. **(B)** SHAP dependence plot of age on AD risk. **(C)** SHAP value distribution of snoring frequency on AD risk. **(D)** SHAP dependence plot of grip strength on AD risk. **(E)** SHAP value distribution of sleep duration on AD risk.

The SHAP dependence plots showed that the frequency of fruit intake was inversely associated with the high-risk state of Alzheimer's disease in a gradient manner. As intake frequency increased, SHAP values gradually decreased, with the lowest SHAP value observed for daily fruit intake, suggesting a strong protective effect (Figure 3A). Age was positively associated with the high-risk state of Alzheimer's disease. As age increased, SHAP values gradually increased, indicating that advancing age is related to a higher risk state of Alzheimer's disease (Figure 3B). Snoring frequency showed a positive gradient association with the high-risk state of Alzheimer's disease. As snoring frequency increased, SHAP values gradually increased, with the highest SHAP value observed for daily snoring (Figure 3C). Average grip strength showed a U-shaped association with the high-risk state of Alzheimer's disease. SHAP values were higher when grip strength was below 10 kg; the lowest SHAP values occurred in the 20–25 kg range, where the protective effect was most pronounced; the protective effect gradually diminished beyond 25 kg, while SHAP values increased again at higher grip strength levels (Figure 3D). Sleep duration exhibited a U-shaped association with the high-risk state of Alzheimer's disease. SHAP values were lowest for 7–8 h of sleep, suggesting that this range corresponds to a lower risk state of Alzheimer's disease, while both too short and too long sleep durations resulted in increased SHAP values (Figure 3E).

## Discussion

Using health survey data from 6,267 community-dwelling adults, we developed three machine learning models—LASSO logistic regression, random forest, and decision tree—to classify AD8-defined high-risk status for Alzheimer's disease, followed by SHapley Additive exPlanations (SHAP) analysis to interpret the best-performing model. Among the three algorithms, random forest showed the highest discriminative performance and was therefore selected for subsequent SHAP analysis. SHAP interpretation identified fruit intake, age, snoring, mean grip strength, and sleep duration as the most influential predictors and further demonstrated non-linear associations, including a U-shaped relationship between sleep duration and AD high-risk status and a non-linear relationship for mean grip strength. Collectively, these findings suggest that explainable machine learning based on routinely collected community health data may provide useful information for community-based risk stratification and targeted cognitive health promotion.

Fruit intake was the most influential modifiable predictor identified by the SHAP analysis. The SHAP dependence plot demonstrated a graded inverse association between fruit consumption frequency and AD high-risk status, with the lowest SHAP values observed among individuals reporting daily intake, suggesting that regular fruit consumption may be associated with a lower risk of Alzheimer's disease. This finding is consistent with previous epidemiological evidence (11). As an essential component of brain-healthy dietary patterns, including the Mediterranean and MIND diets (12, 13), fruits may confer neuroprotection through multiple biological pathways. They are rich in vitamin C, vitamin E, flavonoids, polyphenols, and dietary fiber, all of which possess antioxidant and anti-inflammatory properties and may mitigate neurodegeneration by reducing oxidative stress and modulating neuroinflammatory responses (14–16). In addition, regular fruit consumption contributes to better cardiovascular health by improving blood pressure, glycemic control, and lipid metabolism (17). Because vascular health is closely linked to cognitive function, these benefits may partially explain the lower AD risk associated with higher fruit intake (18). Notably, the SHAP dependence plot further revealed a dose–response pattern, with progressively lower SHAP values as fruit intake frequency increased, indicating that more frequent fruit consumption was associated with a progressively lower likelihood of model-classified AD high-risk status. These findings further support the importance of promoting healthy dietary habits as part of community-based strategies for maintaining cognitive health.

Given that fruit consumption is a modifiable behavior, health education and dietary guidance can be readily implemented at the community level. Therefore, strengthening public awareness campaigns on fruit intake and promoting a balanced diet may serve as key intervention strategies for promoting cognitive health.

Age was another major predictor identified by the SHAP analysis, making an important contribution to model classification. The SHAP dependence plot further demonstrated a progressive increase in AD high-risk status with advancing age, consistent with previous epidemiological evidence (1). Age is widely recognized as the strongest non-modifiable risk factor for Alzheimer's disease. As individuals age, pathological changes—including neuronal loss, synaptic dysfunction, and the accumulation of β-amyloid plaques and tau pathology—gradually develop, ultimately contributing to cognitive decline (19). In addition, aging is accompanied by vascular dysfunction, oxidative stress, chronic low-grade inflammation, and mitochondrial impairment (20), all of which interact to accelerate neurodegenerative processes. The SHAP dependence plot provides a clear visualization of this continuous age-related increase in model-predicted AD high-risk status, highlighting the importance of prioritizing cognitive screening and preventive health management among older adults. Accordingly, comprehensive health promotion strategies targeting modifiable factors—including sleep optimization, healthy dietary habits, and maintenance of physical function—may be valuable components of community-based programs aimed at promoting healthy cognitive aging.

The SHAP dependence plot revealed a U-shaped association between grip strength and model-classified AD8-defined high-risk status. Individuals with grip strength below 10 kg exhibited relatively high SHAP values, whereas the lowest values were observed within the 20–25 kg range. As grip strength increased beyond this range, SHAP values gradually rose again. These findings suggest that the association between grip strength and cognitive health is unlikely to be purely linear and may involve threshold or non-linear effects. Previous studies have consistently reported that lower grip strength is associated with cognitive decline, incident dementia, and increased all-cause mortality (21). Reduced grip strength may reflect sarcopenia, physical inactivity, and impaired neuromuscular function, all of which may contribute to cognitive impairment through decreased neurotrophic signaling, chronic inflammation, and reduced brain plasticity (22). The elevated AD risk observed among individuals with lower grip strength in the present study is consistent with these findings. Notably, our SHAP analysis further indicated a non-linear rather than a linear association, suggesting that progressively greater grip strength did not correspond to a continuously lower predicted AD risk. However, because relatively few participants exhibited very high grip strength and the observed pattern may also have been influenced by residual confounding from age, sex, occupation, and physical activity, this non-linear relationship should be interpreted with caution and confirmed in larger prospective studies. Nevertheless, grip strength measurement is simple, inexpensive, and readily applicable in routine community health assessments. As an objective indicator of physical function, grip strength may serve as a useful complement to community-based cognitive health assessment and risk stratification.

One of the most notable findings of this study was the U-shaped association between sleep duration and model-classified AD high-risk status revealed by the SHAP dependence plot. The lowest SHAP values were observed among individuals reporting 7–8 h of sleep, whereas both shorter and longer sleep durations were associated with progressively higher SHAP values. In addition, snoring frequency demonstrated a positive dose–response association with AD high-risk status, with SHAP values increasing as snoring became more frequent. Together, these findings suggest that both sleep quantity and sleep quality may play important roles in cognitive health. A growing body of evidence indicates that both insufficient and excessive sleep are associated with an increased risk of cognitive decline and dementia (23). The U-shaped relationship observed in our study is consistent with previous epidemiological findings and supports the concept that maintaining an appropriate sleep duration may be more beneficial than simply increasing total sleep time. Short sleep duration may impair the clearance of β-amyloid and tau proteins while promoting neuroinflammation, oxidative stress, and synaptic dysfunction (24). In contrast, prolonged sleep duration may reflect underlying chronic disease, depression, physical inactivity, or early neurodegenerative changes, all of which have been linked to cognitive impairment (25). Frequent snoring, a common manifestation of sleep-disordered breathing, may represent another important indicator of cognitive risk. In particular, habitual snoring often suggests the presence of obstructive sleep apnea (OSA), characterized by recurrent nocturnal hypoxia, sleep fragmentation, and sustained sympathetic activation. These pathophysiological changes can induce oxidative stress, chronic inflammation, cerebrovascular dysfunction, and neuronal injury, thereby contributing to cognitive decline (26). Consistent with these mechanisms, the SHAP dependence plot showed progressively greater contributions of snoring frequency to model-predicted AD high-risk status, indicating that snoring may serve not only as a marker of poor sleep quality but also as a readily available indicator associated with cognitive risk. Because sleep behaviors are highly modifiable, interventions targeting sleep health may represent an effective strategy for community-based cognitive health promotion. Strengthening sleep health education, encouraging regular sleep schedules, and improving the early detection and management of sleep disorders—particularly habitual snoring and obstructive sleep apnea—may contribute to community-based cognitive health promotion and support appropriate referral or further assessment when clinically indicated.

The present findings have several important implications for public health practice. First, the key predictors identified in this study—including fruit intake, sleep duration, snoring, and mean grip strength—are routinely available through community health surveys and standard physical examinations. Unlike neuroimaging, biomarker assessments, or genetic testing, these measures are inexpensive, easily obtainable, and readily scalable, making them potentially useful for community-based risk stratification and cognitive health promotion of Alzheimer's disease. Second, most of these predictors are modifiable through lifestyle or behavioral interventions. Comprehensive health promotion strategies—including improving dietary habits, optimizing sleep duration, strengthening the detection and management of sleep-disordered breathing, and maintaining physical function—may therefore represent potentially relevant approaches to promoting cognitive health among community-dwelling adults. Finally, the non-linear associations identified between sleep duration, mean grip strength, and AD8-defined high-risk status highlight the importance of considering optimal rather than extreme levels of health behaviors and physical function. These findings may inform more targeted and individualized approaches to community-based cognitive health promotion.

This study has several limitations that should be acknowledged. First, owing to its cross-sectional design, only associations between health characteristics and AD high-risk status could be identified, and temporal or causal relationships cannot be inferred. Prospective longitudinal studies are warranted to confirm these findings. Second, AD high-risk status was determined using the AD8 screening scale rather than clinical diagnosis or objective measures such as neuroimaging and biomarkers. Therefore, the findings should be interpreted as AD8-based community risk screening rather than evidence of clinically diagnosed Alzheimer's disease. Finally, the predictors included in the present model were derived primarily from routine community health surveys and standard physical examinations. Potentially informative factors, including genetic susceptibility, blood-based or cerebrospinal fluid biomarkers, and neuroimaging measures, were not incorporated. Future studies should perform external validation and integrate multimodal data with prospective follow-up to further improve model performance and enhance its applicability in community-based cognitive health management.

## Conclusion

Using community-based health survey data, we developed an explainable machine learning model for identifying individuals with AD8-defined high-risk status for Alzheimer's disease. Among the evaluated algorithms, the random forest model demonstrated the highest discriminative performance. SHAP analysis identified age, fruit intake, sleep duration, snoring, and mean grip strength as the most influential predictors of AD high-risk status and further revealed non-linear associations of sleep duration and mean grip strength with model-predicted risk. These findings suggest that explainable machine learning based on routinely collected community health indicators may provide useful information for community-based risk stratification and the development of targeted health promotion and preventive intervention strategies.

## Data Availability

The data analyzed in this study is subject to the following licenses/restrictions: The dataset is not publicly available because it contains sensitive personal health information and is subject to ethical and institutional restrictions designed to protect participant confidentiality. Access to the data may be considered upon reasonable request to the corresponding author, subject to approval by the relevant ethics committee and the data-owning institution. Requests to access these datasets should be directed to Pingjing Wen, wenpj@gxtcmu.edu.cn.
