# Supplementary material for: Explainable machine learning for identifying high-risk Alzheimer's disease and key health characteristics among community-dwelling adults
Source: Front Public Health. 2026 Oct 12;14:1943300. doi: 10.3389/fpubh.2026.1943300 (PMC13696274; doi:10.3389/fpubh.2026.1943300)
Supplement: Supplementary file 1 [file Table_1.docx]

## **Supplementary Table S1**

**Table S1. Final model parameters and classification thresholds**

| Item | Value |
| --- | --- |
| Training set, n | 4,388 |
| Testing set, n | 1,879 |
| Random forest: ntree | 500 |
| Random forest: optimal mtry | 2 |
| Random forest: cross-validated AUC | 0.604 |
| Random forest: class weight, low risk | 0.464 |
| Random forest: class weight, high risk | 1.536 |
| Random forest: Youden cutoff | 0.239 |
| LASSO: optimal λ (lambda.1se) | 0.0207 |
| LASSO: cross-validated AUC | 0.627 |
| LASSO: Youden cutoff | 0.2355 |
| Decision tree: optimal cp | 0.001 |
| Decision tree: cross-validated AUC | 0.528 |
| Decision tree: Youden cutoff | 0.2343 |
| Decision tree: minsplit | 20 |
| Decision tree: maxdepth | 10 |
| Ordinal variable coding | 1 = never, 2 = rarely, 3 = sometimes, 4 = often, 5 = daily |
| SHAP dataset | Testing set |

**Note:** Five-fold cross-validation was performed entirely within the training set. Class weights were calculated as the inverse of class frequencies in the training set and normalized to a mean of 1.
